# Survivors of early childhood trauma: evaluating a two-dimensional diagnostic model of the impact of trauma and neglect

**DOI:** 10.3402/ejpt.v5.21824

**Published:** 2014-04-04

**Authors:** Marleen Wildschut, Willemien Langeland, Jan H. Smit, Nel Draijer

**Affiliations:** 1 GGZ Friesland, Leeuwarden, The Netherlands; 2 Bascous, France; 3 Department of Psychiatry and EMGO Institute, Vrije University Medical Center/GGZinGeest, Amsterdam, The Netherlands; 4 Department of Research, GGZinGeest, Amsterdam, The Netherlands

**Keywords:** trauma-related disorders, personality disorders, early childhood trauma, emotional neglect, treatment indication, diagnostics

## Abstract

**Background:**

A two-dimensional diagnostic model for (complex) trauma-related and personality disorders has been proposed to assess the severity and prognosis of the impact of early childhood trauma and emotional neglect. An important question that awaits empirical examination is whether a distinction between trauma-related disorders and personality disorders reflects reality when focusing on survivors of early childhood trauma. And, is a continuum of trauma diagnoses a correct assumption and, if yes, what does it look like?

**Objective:**

We describe the design of a cross-sectional cohort study evaluating this two-dimensional model of the impact of trauma and neglect. To provide the rationale of our study objectives, we review the existing literature on the impact of early childhood trauma and neglect on trauma-related disorders and personality disorders. Aims of the study are to: (1) quantify the two-dimensional model and test the relation with trauma and neglect; and (2) compare the two study groups.

**Method:**

A total of 200 consecutive patients referred to two specific treatment programs (100 from a personality disorder program and 100 from a trauma-related disorder program) in the north of Holland will be included. Data are collected at the start of treatment. The assessments include all DSM-5 trauma-related and personality disorders, and general psychiatric symptoms, trauma history, and perceived emotional neglect.

**Discussion:**

The results will provide an evaluation of the model and an improvement of the understanding of the relationship between trauma-related disorders and personality disorders and early childhood trauma and emotional neglect. This may improve both diagnostic as well as indication procedures. We will discuss possible strengths and limitations of the design.

Acouple of years ago, across The Netherlands, so-called Top Referent Trauma Centres (TRTCs) were founded. The main goal of these tertiary centers is to improve specialized diagnosis of and treatment for adult survivors of early childhood trauma. The centers were flooded by patients with a wide range of pathology. There is a question to be answered, though. Who are these adult survivors of early childhood trauma in terms of clinical characteristics? And more specifically, do patients with personality disorders belong to this group?

When this inclusion question was raised during meetings on the development of guidelines for assessment and treatment planning of the TRTCs, the discussion focused on patients with borderline personality disorder (BPD). Patients with BPD seem to be the most likely personality disordered survivors of early childhood trauma. However, are these patients to be included in specific treatments within TRTCs? Especially or only when there is a comorbid trauma-related diagnosis, such as posttraumatic stress disorder (PTSD) and/or dissociative disorders? Or do borderlines simply cloud an otherwise pretty clear picture of early-traumatized patients?

In the *Diagnostic and Statistical Manual of Mental Disorders*, Fifth Edition (DSM-5, APA, 2013), trauma-related disorders and personality disorders are still conceptualized as distinct diagnostic categories. When focusing on survivors of early childhood trauma, however, this distinction in diagnostic categories may not make sense. The nature of the problems of survivors of early childhood trauma might be viewed from a problem-oriented as well as a person-oriented approach. Studies to date tend to focus on separate disorders, rather than employing a dimensional model of the impact of trauma and emotional neglect, discussed later, and thus making it difficult to view both diagnostic categories as intertwined when it comes to survivors of early childhood trauma. The current paper presents the design and objectives of our study evaluating a two-dimensional diagnostic model (Draijer, 2003) to examine the clinical characteristics of the two diagnostic categories. The study was designed to address two objectives: the primary aim is to quantify the dimensional model and to test the relation with trauma and emotional neglect, and the secondary aim is to compare the two study groups. To provide the rationale of the study goals and aims, we first briefly review the research on personality disorders and trauma-related disorders in relation to early childhood trauma, findings that have led to the proposal of the dimensional model (Draijer, 2003) that forms the basis of our study. Then problems in defining early childhood trauma are considered. Finally, we present the design of our study to evaluate the dimensional model.

## Rationale of the study

While clinical personality disorders and (complex) trauma-related disorders overlap, the existing research does not reflect this overlap so far and relevant studies to date suffer from a variety of methodological shortcomings (e.g., Fossati, Maddedu, & Maffei, 1999). Many studies have focused on the relationship between BPD and early childhood trauma (e.g., Bandelow et al., 2005; Herman, Perry, & van der Kolk, 1989; Nigg et al., 1991; Silk, Lee, Hill, & Lohr, 1995). Other studies took a broader perspective and focused on the relationship between early childhood trauma and personality disorders in general (e.g., Driessen, Schroeder, Widmann, von Schönfeld, & Schneider, 2006; Weber et al., 2008) as well as specific personality disorders (e.g., Johnson, Smailes, Cohen, Brown, & Bernstein, 2000; Krischer & Sevecke, 2008). However, much of the research done in this area has been limited by design problems, such as the use of different control subjects and different definitions of sexual abuse, the use of unfit study designs, or measures for personality disorders (Fossati et al., 1999). For example, studies tend to measure “personality disorders” in a dimensional way, sometimes in relatively healthy samples, without subjects actually having a clinical diagnose of a personality disorder (e.g., Berenbaum, Thompson, Milanak, Boden, & Bredemeier, 2008; Johnson, Cohen, Brown, Smailes, & Bernstein, 1999). Concerning the instruments used, Allen, Huntoon, and Evans (1999), Johnson, Sheahan, and Chard (2003), and Shea, Zlotnick, and Weisberg (1999) depend on self-report measures for establishing a clinical diagnosis of a personality disorder, whereas such a measure should ideally be used as a screener only. Laporte and Guttman (1996) used psychiatric records of female patients with a discharge diagnosis of personality disorder, which are not standardized and therefore unreliable, to establish a population of women with personality disorders. Finally, methodologically sound studies like the Collaborative Longitudinal Study of Personality Disorders (McGlashan et al., 2000; Zlotnick et al., 2003) focus on certain personality disorders, not all, thus limiting the scope of the study. To our knowledge, no methodologically sound studies are available in which all personality disorders are considered.

Since the 1980s, the study of (complex) PTSD and dissociative disorders, also known as trauma-related disorders, was developed. Traditionally, dissociative disorders have been associated with early childhood trauma (as will be discussed below), while the study of complex PTSD also includes traumatic experiences in adult life.

Based on the DSM-IV PTSD Field Trials, the feasibility of a constellation of trauma-related symptoms not addressed by the PTSD diagnosis, referred to under a variety of names, including complex PTSD (Herman, 1992), complicated PTSD, disorders of extreme stress, and disorders of extreme stress not otherwise specified (Van der Kolk et al., 1996; Van der Kolk, Roth, Pelcovitz, Sunday, & Spinazolla, 2005), was examined. Also, the reliability of a structured interview to measure this symptom constellation was investigated. Finally, this symptom constellation was incorporated into the DSM-IV nomenclature under “associated features of PTSD” (APA, 1994, p. 456). Nine of the 12 symptoms listed under the associated features of PTSD are derived from the complex PTSD theory and constellation based on the DSM-IV PTSD Field Trials (Roth, Newman, Pelcovitz, Van der Kolk, & Mandel, 1997). However, these field trials did not address the comorbidity or overlap with personality disorders (Van der Kolk et al., 2005). On the verge of the fifth edition of DSM, almost 20 years after DSM-IV, there was still much debate about the construct validity of complex PTSD (Herman, 2012, Resick et al., 2012). The conclusion is that there is insufficient evidence to warrant the addition of a complex PTSD diagnosis in the DSM-5. A complex PTSD diagnosis might be added in the upcoming 11th edition of the International Classification of Disorders (ICD-11), though (Cloitre, Garvert, Brewin, Bryant, & Maercker, 2013).

The co-occurrence of dissociative disorders and personality disorders has been investigated among adults in the community. Johnson et al. (2005) found that individuals with personality disorders were substantially more likely than those without personality disorders to have a dissociative disorder. Sar, Akyüz, and Dogan (2007), using a sample of women from a city in a less-industrialized part of Turkey, found that among the group of women with dissociative pathology the psychiatric comorbidity in terms of lifetime PTSD and BPD (the only personality disorder measured in the sample) was significantly higher than for the other study participants. Also, in clinical samples among patients with dissociative disorders, severe personality pathology was found (Boon & Draijer, 1993). Apparently, the two disorders are linked.

Most of the studies in this area consistently focused on early childhood trauma (which in itself, however, is far from a unified concept, as will become clear below) as a possible etiologic mechanism or risk factor, which makes several investigations relatively easy to compare.

Compared to the research on the co-occurrence of dissociative disorders and personality disorders, it is much more difficult to make comparisons between research on personality disorders and on (complex) PTSD. Most research in this area has focused on personality disorders and PTSD without distinguishing between Type I (simple) and Type II (complex) trauma (Terr, 1991) and without distinguishing between early childhood trauma and adult trauma, which is mostly the case in research with samples of combat veterans (e.g., Southwick, Yehuda, & Giller, 1993). Also, the research in this area has focused on several, but not all, of the personality disorders (e.g., Shea et al., 2000). Our study is designed to assess not only all personality disorders but also all trauma-related disorders and to distinguish early childhood trauma from adult trauma and to distinguish trauma (being overwhelmed) from emotional neglect (being unreflected by important others). This design enables us to quantify the dimensional model and to compare the two study groups.

In addition to the paucity of data from studies examining personality disorders and trauma-related disorders in a combined way, studies to date suffer from problems in the choice of a definition of early childhood trauma (Fossati et al., 1999). After the publication of the landmark article “Traumatic antecedents of borderline personality disorder” by Herman and Van der Kolk (1987), the main focus in childhood trauma research has been on sexual abuse alone (e.g., Silk et al., 1995) or combined with physical abuse (e.g., Goldman, D’ Angelo, DeMaso, & Mezzacappa, 1992).

In the 1980s in The Netherlands, attention was drawn to the impact of emotional neglect as well, in addition to and separate from, childhood trauma: neglect increased the risk of occurrence of childhood trauma and also contributed independently to the psychological consequences of childhood trauma (Draijer, 1988). Neglect in the early social environment renders trauma more likely to exert a lasting effect, because the child is unable to either experience or perceive the support of a caregiver able to offset the physiological disturbance caused by trauma (Sabo, 1997).

Emotional neglect has been operationalized in research by Parker, Tupling, and Brown (1979) as (perceived) lack of care and overprotection and is measured with the Parental Bonding Instrument (PBI). Parental dysfunction, that is, parents being emotionally or physically unstable due to mental illness or substance abuse, contributes to emotional neglect. Parental dysfunction is one conceptualization of emotional neglect, referring to the unavailability of parents due to recurrent illness, nervousness, depression, alcohol misuse, or use of sedatives. This measure has been validated by relating it to the lack of parental affection, as measured with the PBI (see Draijer & Langeland, 1999). It was found to be a good indicator of emotional neglect, with the advantage that it refers to factual, observable behavior of parents rather than to more subjective indications of their unavailability or lack of affection.

In their study of childhood trauma and perceived parental dysfunction in the etiology of dissociative symptoms, Draijer and Langeland (1999) found that the severity of dissociative symptoms was best predicted by reported sexual abuse, physical abuse, and maternal dysfunction. Based on their findings, the authors concluded that dissociation, although trauma-related, is neglect-related as well. Johnson et al. (2005), in a literature review of the developmental psychopathology of personality disorders, state that childhood neglect and maladaptive parenting are independently associated with elevated risk for personality disorder even after childhood abuse and parental psychiatric disorders are accounted for.

The literature on personality disorders in general or on specific personality disorders, in particular, shows a tendency to report either on trauma or neglect or on both (e.g., Liotti, Pasquini, & Cirrincione, 2000). Research on personality disorders and PTSD focuses primarily on trauma (e.g., Golier et al., 2003; Shea et al., 2000); only a few researchers pay attention to both trauma and neglect (e.g., Allen et al., 1999).

The literature about dissociative disorders has traditionally focused on the impact of (severe) trauma (Gleaves, May, & Cardena, 2001). However, there is a growing body of research on the effects of neglect on dissociation. Nash, Hulsely, Sexton, Harralson, and Lambert (1993) concluded that abuse was associated with greater use of dissociation, but that this effect was accounted for by family pathology. In a prospective study using a nonclinical, low-income sample of young adults followed from infancy to age 19, Dutra, Bureau, Holmes, Lyubchik, and Lyons-Ruth (2009) found that dissociation in young adulthood was significantly predicted by observed lack of parental responsiveness in infancy, while childhood verbal abuse was the only type of trauma that added to the prediction of dissociation. As described earlier, Draijer and Langeland (1999) concluded that, among psychiatric inpatients, dissociation, although trauma-related, is neglect-related also.

In summary, considering the literature on personality disorders and trauma-related disorders, there seems to be a wide variety in the measurement of personality disorders and trauma-related disorders as well as in the measurement of (early childhood) trauma and neglect. This makes it very difficult to draw conclusions about the relationship between trauma-related disorders—PTSD, complex PTSD, dissociative disorders—and personality disorders based on the existing research. As our study incorporates data collection on all personality disorders, all trauma-related disorders, as well as early childhood trauma and emotional neglect, our study may contribute to an understanding of differential characteristics of patient groups.

## A dimensional model of the impact of trauma and neglect: research questions

**Fig. 1:**
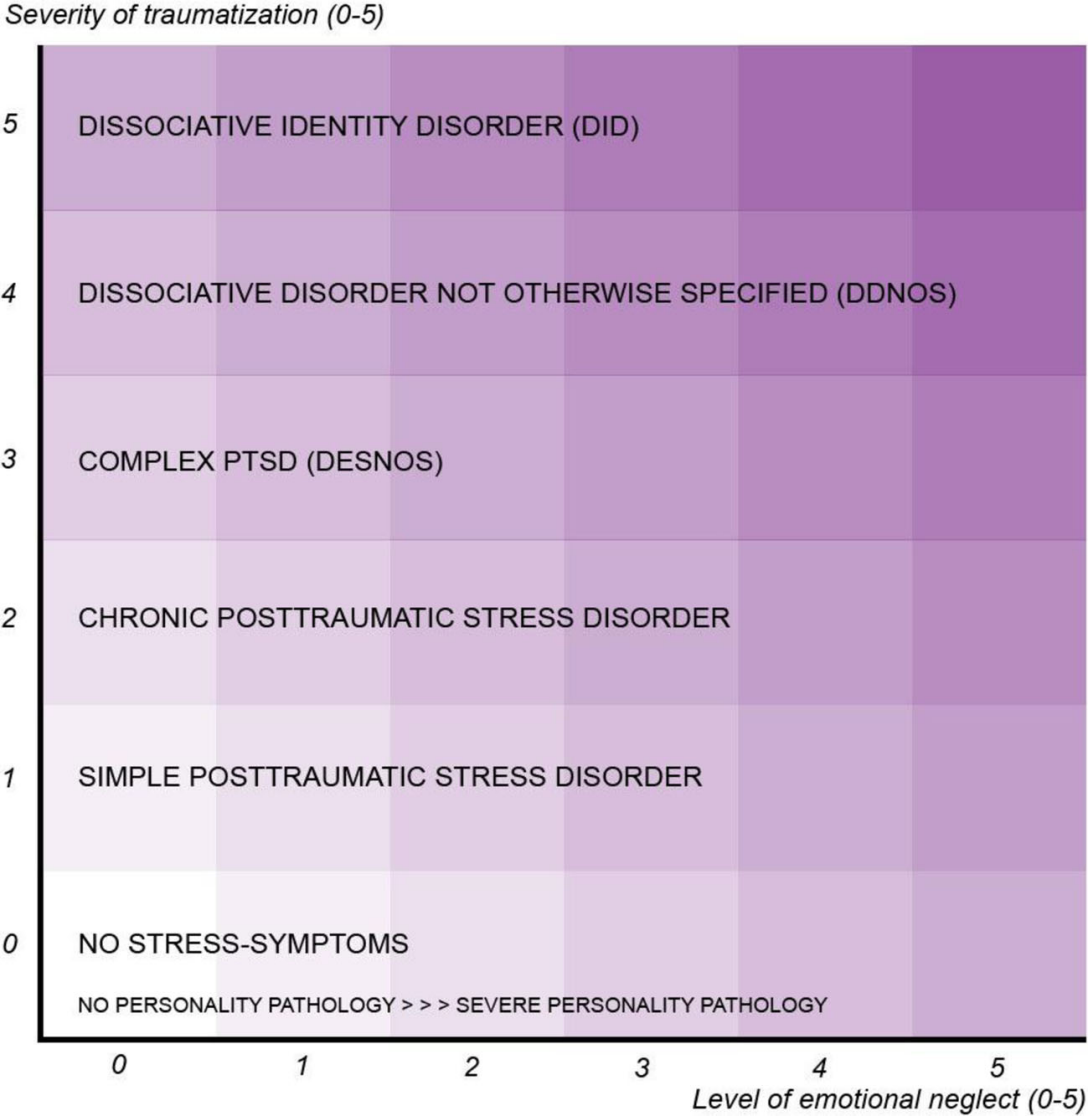
A diagnostic model for the spectrum of trauma-related disorders and personality disorders.

Study findings in the research areas described above have led to the proposal of a two-dimensional diagnostic model (Draijer, 2003; see Fig. 1) that accounts for both the influence of trauma as well as the influence of neglect on the development of trauma-related disorders and personality disorders, respectively. The model was published in The Netherlands and internationally introduced at the annual conference of the International Society for the Study of Trauma and Dissociation (ISSTD) in Washington DC, in 2009. The primary aim of our study is to quantify this dimensional model and to test the relation with trauma and neglect.

Draijer uses two dimensions to “color” the spectrum of trauma-related disorders and personality disorders and to give an indication of their treatability by psychotherapy. The first dimension, situated on the y-axis, consists of the severity of the trauma endured. This severity fluctuates depending on factors such as the age at which the trauma occurred, whether it was physically intrusive, how much force was used, how frequently it occurred, the relationship to the perpetrator, and the number of perpetrators. This dimension is assumed to be related primarily to trauma-related disorders. The second dimension, situated on the x-axis, consists of the severity of emotional neglect or, in other words, the (negative) quality of the early bond with the primary caregivers. This dimension might be related to personality pathology as well as to trauma-related disorders.

The strength of this model is that it incorporates both a varying trauma-related perspective as well as a personality disorder perspective, so every patient can be “located” somewhere in the two-dimensional square. Although the model has shown its clinical relevance already, an important question to be answered is how does the model relate to reality? Is it possible to relate the categorical diagnoses of trauma-related disorders and personality disorders to a diagnostic square, vertically, the severity of trauma and, horizontally, the quality of (perceived) emotional neglect, in a quantified way? Eventually, the square—once validated—could be related to psychotherapeutic treatment response and be helpful in treatment indication: the darker the square, the greater the chance that psychotherapy will not be effective.

## Method

### Participants

To quantify the model, we will study two cohorts—one consisting of consecutively referred patients with a clinical diagnosis of a trauma-related disorder (*N*=100), the other consisting of consecutively referred patients with a clinical diagnosis of a personality disorder (*N*=100)—in the Dutch province of Friesland, north Holland. The assessments are embedded within the local Routine Outcome Monitoring system.

In Friesland, all psychiatric care is divided into diagnostic-driven treatment programs. The patients will be subjected to a similar diagnostic procedure at the start of treatment, using well validated diagnostic instruments designed to measure all DSM-IV trauma-related disorders and personality disorders. Inclusion criteria are: being referred for treatment to either a personality disorders treatment program or a trauma-related disorders treatment program (the latter program is the TRTC-program, aimed specifically at adult survivors of prolonged early childhood trauma). The exclusion criterion is insufficient mastery of the Dutch language.

### Study design

Considering the number of patients referred to both treatment programs, it will be possible to include all patients consecutively referred to the trauma-related disorders program (which only provides outpatient care) during a time period of 1.5 years. Due to the larger set-up of the personality disorders program (which also has inpatient facilities), we will include all consecutively referred patients given a time-frame of multiple months in one department and then move on to the next. The study protocol has been reviewed and approved by the Medical Ethics Committee of the Stichting Medisch-Ethische Toetsingscommissie Instellingen Geestelijke Gezondheidszorg (METiGG; registration no. 11.121).

### Procedure

Patients are contacted by an interviewer after admission to treatment in one of the two specific treatment programs. If patients agree to participate, informed consent is obtained and patients are scheduled for their first appointment with the interviewer. The patient decides how many appointments it takes to complete the whole battery of assessment instruments (usually it takes four appointments, since the whole battery takes about 10 hours to administer) and most patients take the self-report questionnaires home (though the possibility to have assistance when filling in the questionnaires is offered). All interviewers are trained and supervised psychologists. Most interviews are videotaped and evaluated during supervision. After the administration of all instruments, the patient is provided with the results in the form of a psychological report.

### Assessments

All assessments include standardized measures. Table 1 provides an overview of the instruments that comprise the whole assessment. We will describe the instruments in more detail later.

**Table 1:** Overview of assessment measures

Construct	Instrument	Interview or self-report
Trauma	STI	Interview
Parental bonding/emotional neglect	PBI	Self-report
General psychopathology	SCL-90-R	Self-report
	IDS	Self-report
	BAI	Self-report
	DES	Self-report
Trauma-related diagnoses	CAPS	Interview
	SIDES	Interview
	SCID-D	Interview
Personality disorder diagnoses	SIDP-IV	Interview
Dimensional character problems	SIPP-118	Self-report
	Young Schema-Questionnaire	Self-report
Big Five personality traits	NEO-PI-R	Self-report

*Note*. Structured Trauma Interview (STI; Draijer, 1989); Parental Bonding Instrument (PBI; Parker et al., 1979); Symptom Checklist-90-Revised (SCL-90-R; Arrindell & Ettema, 1986); Inventory of Depressive Symptomatology (IDS; Rush et al., 1996); Beck Anxiety Inventory (BAI; Steer & Beck, 1997); Dissociative Experiences Scale (DES; Bernstein & Putnam, 1986); Clinician Administered PTSD Scale (CAPS; Blake et al., 1995); Structured Interview for Disorders of Extreme Stress (SIDES; Pelcovitz et al., 1997); Structured Interview for DSM-IV Dissociative Disorders (SCID-D-R; Steinberg, 2000); Structured Interview for DSM Personality Disorders (SIDP-IV; Pfohl et al., 1995); Severity Indices of Personality Problems (SIPP-118; Verheul et al., 2008); Young Schema Questionnaire (Rijkeboer et al., 2005); NEO-PI-R (Costa & McCrae, 1995).

For the measurement of traumatic experiences (loss of primary caretakers, witnessing violence between primary caretakers, physical abuse, sexual abuse, and other shocking events during child- and adulthood), the Structured Trauma Interview (STI; Draijer, 1989) will be used. Neglect will be measured with the (PBI; Parker et al., 1979), which allows five types of parental bonding to be examined, based on two dimensions: care and overprotection. To assess trauma-related disorders and personality disorders in a reliable fashion, we choose to use structured psychiatric interviews, including the Structured Interview for DSM-IV Dissociative Disorders (SCID-D-R; Steinberg, 2000), the Clinician Administered PTSD Scale (CAPS; Blake et al., 1995), the Structured Interview for Disorders of Extreme Stress (SIDES; Pelcovitz et al., 1997) and the Structured Interview for DSM Personality Disorders (SIDP-IV; Pfohl, Blum, & Zimmerman, 1995).

Anticipating DSM-5, personality pathology will also be measured in a dimensional way. Patients will be evaluated considering their level of (mal)adaptive personality functioning, using the Severity Indices of Personality Problems (SIPP-118; Verheul et al., 2008), schemas (as in general themes or patterns, which consist of memories, emotions, cognitions and physical experiences related to the self and to relationships with others; Rijkeboer, Van den Bergh, & Van den Bout, 2005), and general personality traits (NEO-PI-R; Costa & McCrae, 1995).

General psychopathology will also be taken into account, using the Symptom Checklist-90-Revised (SCL-90-R; Arrindell & Ettema, 1986), the Inventory of Depressive Symptomatology (IDS; Rush, Gullion, Basco, Jarrett, & Trivedi, 1996), and the Beck Anxiety Inventory (BAI; Steer & Beck, 1997). Finally, dissociative symptoms will be measured using the Dissociative Experiences Scale (DES; Bernstein & Putnam, 1986).

### Data analysis

The purpose is first to test the difference in demographics, trauma-related and clinical characteristics between the two groups of patients (using Chi-square or *t*- or *F*-tests); second to test the relationship between trauma severity and trauma-related diagnosis (using *t-* or *F*-tests), as well as the relationship between the severity of personality pathology/maladaptive functioning and neglect (using Pearson's r); and finally, to localize the patients within the diagnostic square (using cluster analysis). To achieve the latter, an estimated 200 patients (100 in each group) is required.

## Discussion

The relationship between trauma-related disorders, personality disorders, and early childhood trauma and neglect is far from clear. To be able to determine which patients will be eligible for treatment in tertiary trauma centers for survivors of early childhood trauma like the Dutch TRTCs, research is needed on how the relationship between these disorders must be understood. In the absence of such knowledge, early childhood trauma survivors with personality disorders run the risk of being “left out” when it comes to specialized treatment. In this light, it is important to know if a trauma diagnosis continuum is a correct assumption and, if yes, what it looks like.

The study design seems to be relatively straightforward; however, many practical and logistical challenges need to be addressed. The first author is locally responsible for the activation of the study protocol. The study is embedded in the daily clinical routine in the trauma-related disorders treatment program (the TRTC-program). Because of this routine, trained staff and performing of measurements is already arranged and realistic predictions can be made about inclusion rates, as well as realistic estimates of the time assessments require. However, for the purpose of the study, this daily routine needs to be temporarily incorporated in the personality disorders treatment program as well. Moreover, this treatment program is relatively large and consists of several units providing both inpatient and outpatient care, while the TRTC-program consists of only one unit, providing outpatient care. As mentioned before, we will include all consecutively referred patients given a time-frame of multiple months in one department and then move on to the next. Because there are six departments, it will not be possible to cover all. In order to discuss the representativeness of our study sample in the personality disorders program, we will need to compare demographic and clinical characteristics to those of all patients in a personality disorders program in the organization.

Because the personality disorders treatment program also offers inpatient care, we have decided to include patients from this department in our sample, since it might be that the patients who are affected most severely by their personality disorder, might also be the ones with the most severe trauma-related pathology.

An important limitation that may affect the results of the proposed study is that the interviewers are not blind to which patient is referred to which program. We anticipated for this difficulty by providing frequent personal supervision by a senior clinical psychologist and regular meetings with the whole research group during which video-taped interviews are discussed.

Another possible limitation is that patients in the personality disorders program are offered the assessment battery as part of the current research and not as part of a regular diagnostic procedure, as is the case for patients in the trauma-related program. This might lead to differences in participants versus non-participants in the two groups. As mentioned earlier, we will compare demographic and clinical characteristics of participants to those of all patients in a personality disorders program in the organization to evaluate the representativeness of our study sample.

A particular strength of this study is that it is unique in the elaborate way in which traumatization and pathology are being investigated. An improvement of the understanding of the relationship between trauma-related disorders and personality disorders and the role early childhood trauma plays in them could lead to a more accurate decision-making policy considering the targeted group of the TRTCs and clarify the view on both disorders in general.
